# Genes functioned in kleptoplastids of *Dinophysis* are derived from haptophytes rather than from cryptophytes

**DOI:** 10.1038/s41598-019-45326-5

**Published:** 2019-06-21

**Authors:** Yuki Hongo, Akinori Yabuki, Katsunori Fujikura, Satoshi Nagai

**Affiliations:** 1 Department of Research Center for Bioinformatics and Biosciences, National Research Institute of Fisheries Science, Japan Fisheries Research and Education Agency, 2-12-4 Fukuura, Kanazawa, Yokohama, Kanagawa 236-8648 Japan; 2 Department of Marine Biodiversity and Environmental Assessment Research Center, Japan Agency for Marine-Earth Science and Technology, 2-15 Natsushima-cho, Yokosuka, Kanagawa 237-0061 Japan

**Keywords:** Evolutionary theory, Molecular evolution

## Abstract

Toxic dinoflagellates belonging to the genus *Dinophysis* acquire plastids indirectly from cryptophytes through the consumption of the ciliate *Mesodinium rubrum*. *Dinophysis acuminata* harbours three genes encoding plastid-related proteins, which are thought to have originated from fucoxanthin dinoflagellates, haptophytes and cryptophytes via lateral gene transfer (LGT). Here, we investigate the origin of these plastid proteins via RNA sequencing of species related to *D. fortii*. We identified 58 gene products involved in porphyrin, chlorophyll, isoprenoid and carotenoid biosyntheses as well as in photosynthesis. Phylogenetic analysis revealed that the genes associated with chlorophyll and carotenoid biosyntheses and photosynthesis originated from fucoxanthin dinoflagellates, haptophytes, chlorarachniophytes, cyanobacteria and cryptophytes. Furthermore, nine genes were laterally transferred from fucoxanthin dinoflagellates, whose plastids were derived from haptophytes. Notably, transcription levels of different plastid protein isoforms varied significantly. Based on these findings, we put forth a novel hypothesis regarding the evolution of *Dinophysis* plastids that ancestral *Dinophysis* species acquired plastids from haptophytes or fucoxanthin dinoflagellates, whereas LGT from cryptophytes occurred more recently. Therefore, the evolutionary convergence of genes following LGT may be unlikely in most cases.

## Introduction

Plastids in photosynthetic dinoflagellates are classified into five types according to their origin^1^. Plastids in most photosynthetic dinoflagellates are bound by three membranes and contain peridinin as the major carotenoid. Although peridinin is considered to have originated from an endosymbiotic red alga^1^, this hypothesis remains controversial^2^. Some photosynthetic dinoflagellates possess plastids originated from other eukaryotic alga. Such occasional plastid replacements have reportedly occurred in several dinoflagellate lineages, resulting in these dinoflagellates possessing plastids originated from haptophytes^3^, stramenopiles^4^, chlorophytes^5^ or cryptophytes^6^. Dinoflagellates with plastids derived from haptophytes contain 19′-hexanoyloxy-fucoxanthin (fucoxanthin) as the major carotenoid^3^. Initial plastid acquisition possibly occurs through the predator–prey interactions, which result in the establishment of a permanent plastid likely through gene loss and lateral gene transfer (LGT) from the plastid to the host cell nucleus^7,8^. Moreover, plastids in some dinoflagellates (e.g. *Dinophysis* spp. and *Nusuttodinium* spp.) are derived from their photosynthetic prey through kleptoplasty^9–11^, which is believed to have been the driving force behind the evolutionary transition towards the establishment of a permanent plastid.

*Dinophysis* spp. can serve as potential models for studying plastid establishment since they acquire plastids from their ciliate prey *Mesodinium rubrum*^12^, which itself derives plastids from its cryptophyte prey^9,13^. Therefore, *Dinophysis* spp. acquire plastids of cryptophyte origin (kleptoplastids). Although *Dinophysis* spp. primarily depend on their prey for nutrition, they can survive for several months without feeding by relying on photosynthesis through kleptoplastids^14,15^. However, unlike permanent plastids, kleptoplastids in *Dinophysis* spp. are not maintained for a sufficiently long-term and eventually need to be replenished via the consumption of more prey. Expression of genes related to cryptophyte plastid function and maintenance is lower in *D. acuminata* than in completely phototrophic algae with permanent plastids^16^, suggesting that *D. acuminata* cannot establish permanent plastids. However, products of at least five genes are reportedly transported to kleptoplastids, three of which are acquired by LGT from fucoxanthin dinoflagellates, haptophytes and cryptophytes^16^. Furthermore, additional genes that likely function in plastids have been reported^17^, although their phylogenetic origins have not been analysed in detail. Studies conducted to date have focused on *D. acuminata* alone, and the extent of dominance of laterally transferred genes in the kleptoplastids of *Dinophysis* remains unknown.

In the present study, we sequenced *D. fortii* transcripts and identified proteins that are generally considered to functions in plastids. The origins of both newly identified and known *D. acuminata* proteins were analysed through phylogenetic studies. Two or more isoforms of the same *D. fortii* protein were examined, and their transcript levels were compared. The findings of this study shed light on the evolutionary transition towards plastid retention in *Dinophysis*.

## Results

### Genes expressed in kleptoplastid-retaining *D. fortii*

Sequencing of cDNA libraries of the cryptophyte alga *Teleaulax amphioxeia*, the ciliate *M. rubrum* and the dinoflagellate *D. fortii* using NextSeq 500 (Illumina Inc., San Diego, CA, USA) yielded a total of 44–110 million reads per species, which were deposited in the DNA Data Bank of Japan (DDBJ) Sequence Read Archive under accession numbers DRX131336–DRX131339, DRX131340–DRX131341 and DRX131342–DRX131343, respectively. Once the adapter and low-quality sequences were trimmed, the remaining reads from each pair of libraries were assembled by Trinity^18^ into 132,239, 144,278 and 217,120 contigs for *T. amphioxeia*, *M. rubrum* and *D. fortii*, respectively (Supplementary Table 1). Removal of the prey sequences from the assembled contigs for *D. fortii* yielded 185,121 contigs as *D. fortii*-derived sequences. Open reading frames (ORFs) of >300 bp were extracted from 122,676 of the assembled *D. fortii* contigs and translated into 423,018 amino acid sequences. Following the clustering of redundant amino acids with up to 95% homology, 372,783 distinct amino acid sequences were obtained (Supplementary Table 1), which were used for sequence homology searches. Overall, 59,907 (16.1%) and 61,878 (16.6%) amino acid sequences showed significant similarities (e-value < 1e^–3^) to protein sequences in the non-redundant proteins (nr) and UniRef90 databases, respectively. Moreover, 3,365 gene ontology (GO) numbers were assigned to 39,850 amino acid sequences (10.7%), and 711 enzyme commission (EC) numbers were assigned to 10,328 amino acid sequences (2.8%) (Supplementary Table 1).

Based on the assigned EC numbers and annotated descriptions, 58 of the amino acid sequences were found to be related to isoprenoid, carotenoid, porphyrin and chlorophyll biosyntheses as well as to photosynthesis; all sequences were registered with DDBJ as transcriptome shotgun assembly (TSA) sequences (Supplementary Table 2). High-resolution phylogenetic trees revealed that 12 *D. fortii* enzymes originated from other organisms, while another 13 originated from peridinin dinoflagellates. Of note, in phylogenetic trees, almost all proteins identified protein in *D. fortii* branched with those in *D. acuminata* with high statistical support and genes of both species shared almost the same evolutionary backgrounds.

### Porphyrin and chlorophyll biosynthesis genes

The phylogenetic trees indicated that the following six porphyrin biosynthetic enzymes originated from peridinin dinoflagellate: glutamate-tRNA ligase, glutamyl-tRNA reductase (HemA), delta-aminolevulinate dehydratase (HemB), uroporphyrinogen decarboxylase (HemE), coproporphyrinogen oxidase (HemF) and protoporphyrinogen oxidase (HemY) (Supplementary Fig. 1). Moreover, ferrochelatase (HemH) was clustered with peridinin dinoflagellates, although as a part of the delta-proteobacteria clade. There was insufficient support to resolve the phylogenetic relationships of glutamate-1-semialdehyde 2,1-aminomutase (HemL), hydroxymethylbilane synthase (HemC) and uroporphyrinogen-III synthase (HemD).

**Figure 1:**
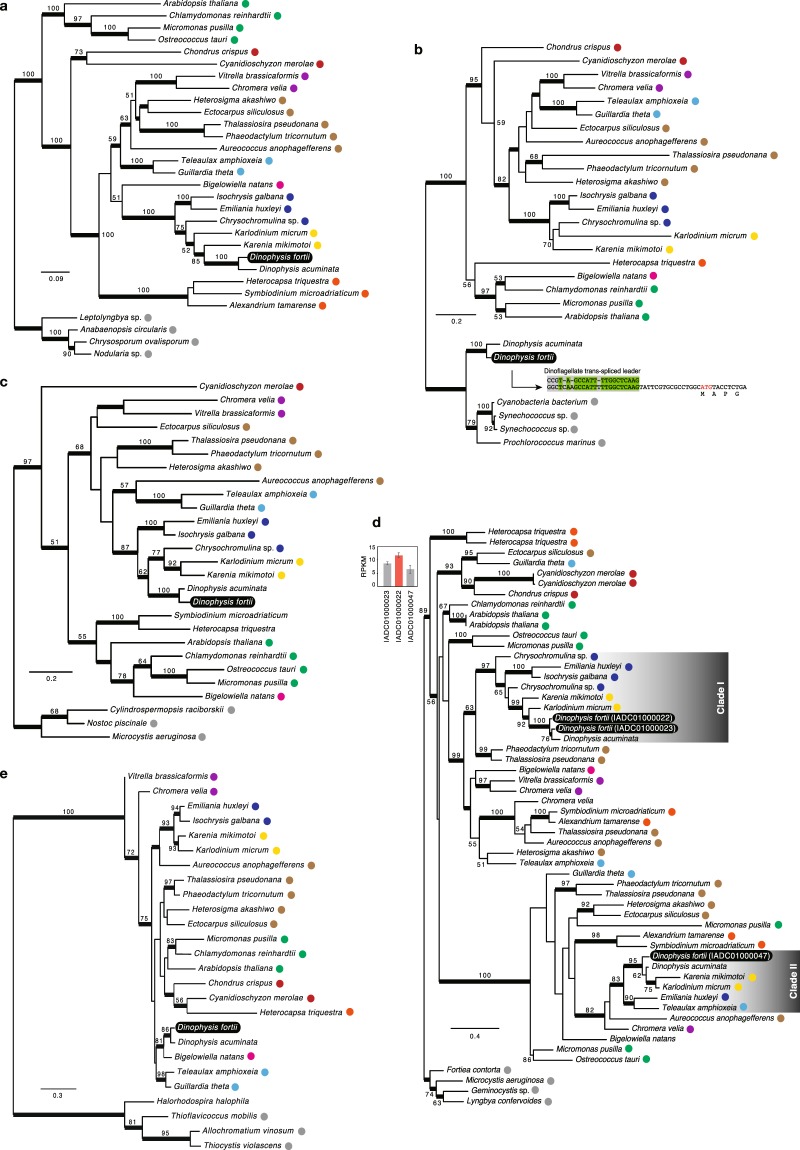
Maximum-likelihood trees of (**a**) ChlH, (**b**) ChlD, (**c**) ChlM, (**d**) POR and (**e**) ChlG. Bootstrap values ≥50% are shown. Nodes supported by Bayesian posterior probabilities ≥0.95 are highlighted with bold lines. Where two or more isoforms were identified, transcription levels are shown in the inset bar chart in the top left corner and the isoform with the highest transcription level is highlighted with a red bar in the bar chart. Transcriptome shotgun assembly (TSA) accession numbers are shown in parentheses. The alignment of the dinoflagellate splice leader sequence and 5′-end of the *chlD* mRNA sequence is shown in (**b**). Coloured circles indicate proteins of peridinin dinoflagellates (orange), fucoxanthin dinoflagellates (yellow), *Haptophyta* (blue), *Chlorarachniophyta* (magenta), *Cryptophyta* (light blue), *Chromerida* (purple), *Rhodophyta* (red), *Stramenopiles* (brown), *Viridiplantae* (green) and *Bacteria* (grey). Proteins of *Dinophysis fortii* are presented on black background with white font.

Regarding chlorophyll biosynthetic enzymes, magnesium-protoporphyrin IX chelatase comprised three subunits (H, D and I). Subunit H (ChlH) of *Dinophysis* was clustered with that of the fucoxanthin dinoflagellate *Karenia mikimotoi*, with moderate statistical support [bootstrap probability (BP) = 85%, Bayesian posterior probability (BPP) = 1.00; Fig. 1a]. Moreover, both subunits were clustered with those of *Karlodinium micrum* and haptophytes, and their monophyly was completely supported (Fig. 1a). Meanwhile, subunit D (ChlD) of *Dinophysis* was clustered with that of cyanobacteria (BP = 100%, BPP = 1.00), and its trans-spliced leader sequence was attached to that of the 5′ end of *D. fortii* mRNA, albeit with six mismatches (Fig. 1b). Magnesium-protoporphyrin IX methyltransferase (ChlM) of *Dinophysis* formed a clade with that of fucoxanthin dinoflagellates and haptophytes, with moderate statistical support (BP = 87%, BPP = 1.00; Fig. 1c). Two distinct isoforms of protochlorophyllide oxidoreductase (POR) were identified in *Dinophysis*, both of which were clustered with those of fucoxanthin dinoflagellates (BP = 99%, BPP = 1.00 in clade I, BP = 95%, BPP = 1.00 in clade II; Fig. 1d). Moreover, one isoform was clustered with haptophyte isoforms (BP = 97%, BPP = 1.00; clade I), while the other with *E. huxleyi* and *T. amphioxeia* isoforms, with moderate statistical support (BP = 83%, BPP = 1.00; clades II). The expression of one POR isoform of *D. fortii* in clade I was significantly higher than that of the other isoforms (one-way analysis of variance [ANOVA], P < 0.05; Fig. 1d). Finally, chlorophyll synthase (ChlG) of *Dinophysis* branched with that of the chlorarachniophyte *Bigelowiella natans*, with moderate statistical support (BP = 81%, BPP = 1.00; Fig. 1e).

### Isoprenoid and carotenoid biosynthesis genes

**Figure 2:**
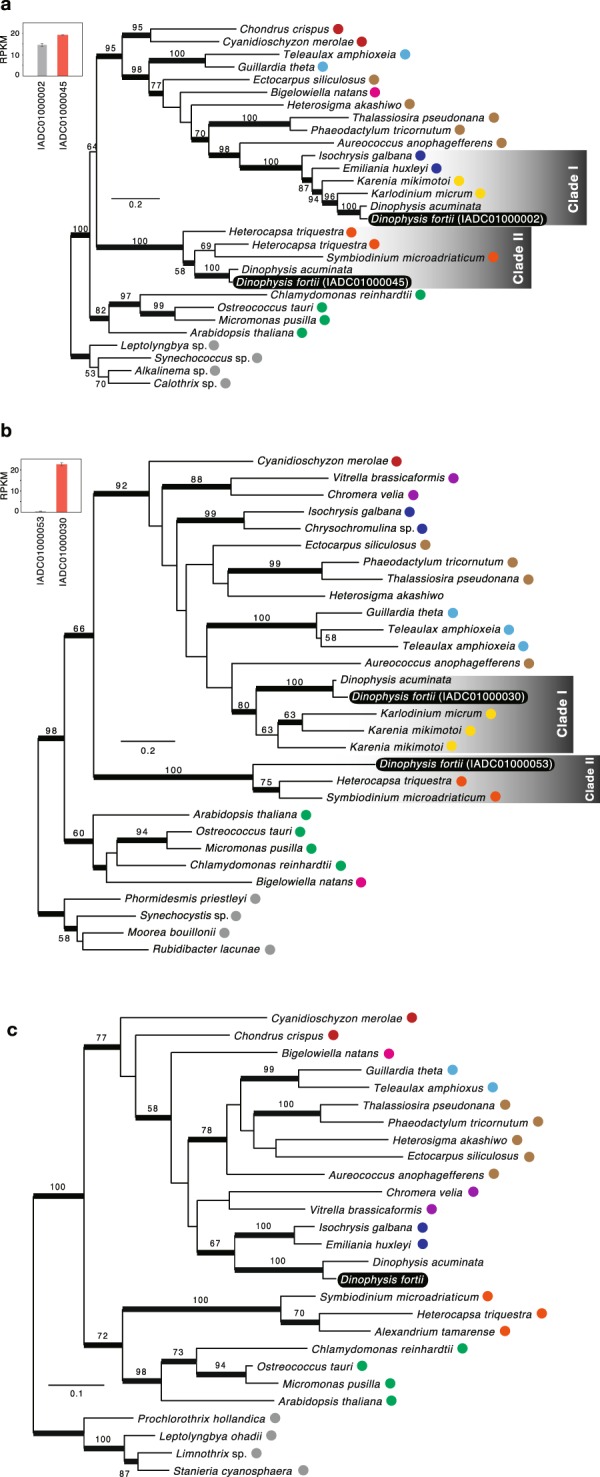
Maximum-likelihood trees of (**a**) IspH, (**b**) phytoene synthase and (**c**) ChlP. Bootstrap values ≥50% are shown. Nodes supported by Bayesian posterior probabilities ≥0.95 are highlighted with bold lines. Where two or more isoforms were identified, transcription levels are shown in the inset bar chart in the top left corner, and the isoform with the highest transcription level is highlighted with a red bar in the bar chart. Transcriptome shotgun assembly (TSA) accession numbers are shown in parentheses. Coloured circles indicate proteins from the same organisms as described in Fig. 1.

Two isoforms each of the enzymes 4-hydroxy-3-methylbut-2-en-1-yl diphosphate reductase (IspH) and phytoene synthase, which are related to isoprenoid and carotenoid biosyntheses (Fig. 2a,b), and one isoform of phytoene synthase was identified in *D. acuminata*. One isoform of *Dinophysis* IspH in clade I was tightly clustered with that of fucoxanthin dinoflagellates (BP = 94%, BPP = 1.00) and with that of haptophytes (BP = 100%, BPP = 1.00; clade I). The other isoforms of *Dinophysis* IspH was tightly clustered with that of peridinin dinoflagellates (BP = 100%, BPP = 1.00; clade II). The expression of *D. fortii* isoform in clade II was significantly higher than that of the isoform in clade I (*t*-test, P < 0.05; Fig. 2a). Similarly, the *Dinophysis* phytoene synthase isoform in clade I was clustered with that of fucoxanthin dinoflagellate isoform, with moderate statistical support (BP = 80%, BPP = 0.99; clade I), whereas the other isoform in clade II was clustered with peridinin dinoflagellates (BP = 100%, BPP = 1.00; clade II). The expression of *D. fortii* isoform in clade I was significantly higher than that of *D. fortii* isoform in clade II (*t*-test, P < 0.05; Fig. 2b). Geranylgeranyl reductase (ChlP) of *Dinophysis*, which produces phytyl diphosphate for chlorophyll biosynthesis, was clustered with that of haptophytes, with weak statistical support (BP = 67%, BPP = 1.00), but was distinct from that of peridinin dinoflagellates (Fig. 2c). Since the fucoxanthin dinoflagellate sequences were not available, the relationship among those could not be elucidated.

Other enzymes related to isoprenoid biosynthesis in *Dinophysis*, including 1-deoxy-D-xylulose-5-phosphate synthase (DXS), 1-deoxy-D-xylulose-5-phosphate reductoisomerase (IspC), 2-C-methyl-D-erythritol 4-phosphate cytidylyltransferase (IspD), 4-(cytidine 5′-diphospho)-2-C-methyl-D-erythritol kinase (IspE), 2-C-methyl-D-erythritol 2,4-cyclodiphosphate synthase (IspF) and 4-hydroxy-3-methylbut-2-en-1-yl diphosphate synthase (IspG) were estimated to have originated from peridinin dinoflagellates (Supplementary Fig. 2). There was insufficient support to resolve the phylogenetic relationships of the two isoforms of farnesyl-diphosphate synthase (FDSP), which is related to carotenoid biosynthesis.

### Photosynthesis genes

**Figure 3:**
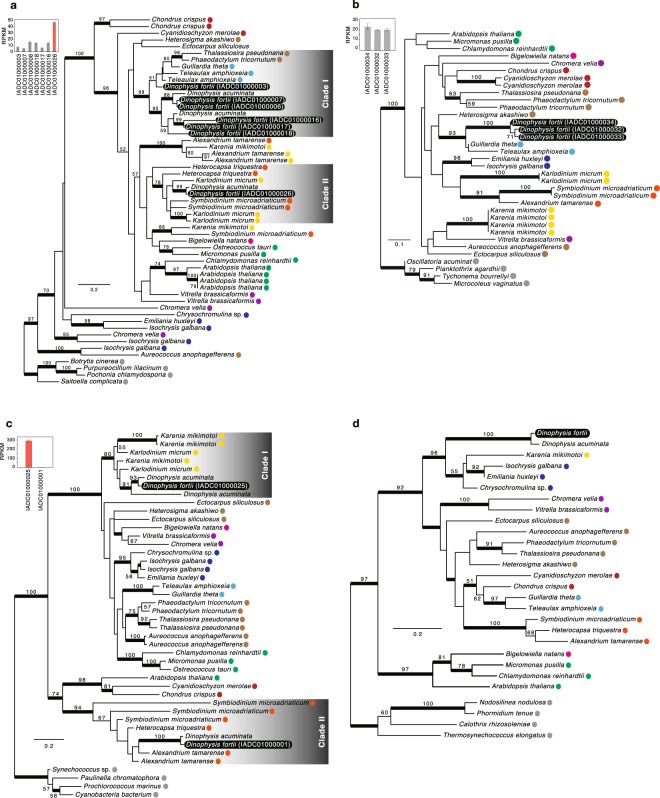
Maximum-likelihood trees of (**a**) ascorbate peroxidase, (**b**) PetC, (**c**) PetH and (**d**) PsbO. Bootstrap values ≥50% are shown. Nodes supported by Bayesian posterior probabilities ≥0.95 are highlighted with bold lines. Where two or more isoforms were identified, transcription levels are shown in the inset bar chart in the top left corner, and the isoform with the highest transcription level is highlighted with a red bar in the bar chart. Transcriptome shotgun assembly (TSA) accession numbers are provided in parentheses. Coloured circles indicate proteins from the same organisms as described in Fig. 1.

Seven and three isoforms of ascorbate peroxidase were identified in *D. fortii*, and *D. acuminata*, respectively. Five and two of the *D. fortii* and *D. acuminata* isoforms, respectively, were clustered in clade I, with moderate statistical support (BP = 90%, BPP = 1.00), which also comprised another *D. fortii* isoform as well as isoforms of cryptophytes *Guillardia theta* and *T. amphioxeia* (BP = 89%, BPP = 1.00). The remaining isoforms of *Dinophysis* were clustered with isoforms of fucoxanthin and peridinin dinoflagellates, although the monophyly was not well supported (BP = 43%, BPP = 0.55; clade II in Fig. 3a). The expression of *D. fortii* isoform in clade II (IADC01000026) was the highest among the seven isoforms, and differences were statistically significant (one-way ANOVA, P < 0.01; Fig. 3a). Three isoforms of cytochrome b6/f complex iron-sulfur subunit (PetC) were identified in *D. fortii*, but none in *D. acuminata*. These isoforms branched together (BP = 100%, BPP = 1.00) and were tightly clustered with cryptophyte isoforms (BP = 93%, BPP = 1.00; Fig. 3b). However, there were no significant differences in the expression levels of these isoforms (one-way ANOVA, P > 0.05; Fig. 3b). Two ferredoxin-NADP( + ) reductase (PetH) isoforms were identified in *Dinophysis*. One isoform was clustered with fucoxanthin dinoflagellate isoforms, with moderate statistical support (BP = 80%, BPP = 1.00; clade I in Fig. 3c), while the remaining isoform was clustered with peridinin dinoflagellate isoforms, with strong statistical support (BP = 94%, BPP = 1.00; clade II in Fig. 3c). The expression of *D. fortii* isoform in clade I was significantly higher than that of isoform in clade II (*t*-test, P < 0.01; Fig. 3c). Finally, the oxygen-evolving enhancer protein (PsbO) of *Dinophysis* was tightly clustered with that of fucoxanthin dinoflagellates and haptophytes (BP = 96%, BPP = 1.00; Fig. 3d).

## Discussion

In this study, we examined the origins of genes encoding *D. fortii* proteins, which are involved in the biosyntheses of porphyrins, chlorophylls and isoprenoids as well as in photosynthesis. We identified 58 proteins involved in these processes, 30 of which originated from peridinin dinoflagellates, 21 from other species via LGT and the origin of the remaining 7 could not be identified. Our findings indicate that the mosaic origin of plastid genes may be a common characteristic of *Dinophysis* spp. and that LGT occurred in common ancestral species of *D. fortii* and *D. acuminata*. Moreover, gene replacement may have occurred, followed by LGT, which is rather rare in some pathways. All proteins involved in porphyrin and isoprenoid biosyntheses appear to have originated from peridinin dinoflagellates, although the phylogenies of three of these proteins (HemL, C and D) could not be resolved in the present study (Supplementary Fig. 1). In addition, HemH originated from peridinin dinoflagellates, although it formed a cluster with the HemH of red algae and delta-proteobacteria (Supplementary Fig. 1j). According to a previous phylogenetic analysis, HemH originated from proteobacteria^19^. Thus, the ancestral species of the peridinin dinoflagellates likely obtained HemH from red algae.

Genes involved in porphyrin and isoprenoid biosyntheses are essential because they produce the chlorophyll backbone as well as haem, which acts as the prosthetic group of cytochromes, catalases and peroxidases during porphyrin biosynthesis^20^ and as a backbone for steroids, sterol and carotenoids in isoprenoid biosynthesis. Thus, the genes involved in essential pathways may be highly conserved and unlikely to be replaced by genes of other origins via LGT. Therefore, *Dinophysis* may possess conserved proteins derived from peridinin dinoflagellate, which are involved in essential biosynthetic pathways. However, *ChlH*, *ChlM* and *POR*, which are involved in chlorophyll biosynthesis following porphyrin biosynthesis, originated from fucoxanthin dinoflagellates, whereas *ChlG* and *ChlD* originated from chlorarachniophytes and cyanobacteria, respectively. The phylogenetic tree indicated that the gene encoding ChlD, a subunit of magnesium-protoporphyrin IX chelatase, was transcribed with a trans-spliced leader sequence in *D. fortii*, whereas the ChlD of *Dinophysis* was derived from cyanobacteria via LGT. (Fig. 1b). Thus, genes involved in chlorophyll biosynthesis appear to have originated from organisms different from which genes involved in porphyrin and isoprenoid biosyntheses were derived from. *Dinophysis* spp. contain 59–221 times higher volumes of chlorophyll a (Chl a) per cell than *T. amphioxeia*; thus, Chl a may be synthesised even in *Dinophysis* cells^21^. However, because *ChlE*/*ChlA* and *DVR* involved in chlorophyll biosynthesis were not identified in the present study, the assumption of additional Chl a biosynthesis in *Dinophysis* cells is not supported by our data. If *ChlE*/*ChlA* and *DVR* are not transcribed, Mg-protoporphyrin IX 13-methyl ester, which is produced by ChlM from Mg-protoporphyrin IX, may accumulate in the kleptoplastids. The accumulation of Mg-protoporphyrin IX 13-methyl ester and/or Mg-protoporphyrin IX regulates chloroplast development via chloroplast signaling mediated by nuclear genes^22–24^; such partial Chl a biosynthetic pathway may play some role in the regulation of kleptoplastid development.

During the final step of isoprenoid biosynthesis, IspH produces isopentenyl diphosphate (IPP), two isoforms of which were detected in this study. One of these isoforms originated from peridinin dinoflagellates, whereas the other originated from fucoxanthin dinoflagellates (Fig. 2a). Moreover, phytoene synthase, which is involved in carotenoid biosynthesis occurring behind isoprenoid biosynthesis, was identified as having two isoforms that originated from peridinin and fucoxanthin dinoflagellates (Fig. 2b). Interestingly, the highest phytoene synthase level was produced by the gene of fucoxanthin dinoflagellate origin, whereas the highest IspH level was produced by the gene of peridinin dinoflagellate origin (Fig. 2a,b). Thus, the genes of different origins are likely not evolutionarily converged. *Dinophysis* spp. and their prey contain alloxanthin as a major carotenoid^21^. Therefore, *D. fortii* may have deviated from using peridinin to other carotenoids since the usage of different origin of genes is controlled by the regulation of their transcription.

Among the genes involved in photosynthesis, six ascorbate peroxidase isoforms were likely derived from cryptophytes and one from peridinin dinoflagellates, which was highly transcribed (Fig. 3a). Thus, the protein encoded by the gene of peridinin dinoflagellate origin may play predominant functions in *Dinophysis* spp. In addition, three *PetC* isoforms originated from cryptophytes (Fig. 3b), suggesting that *PetC* is acquired via LGT and complements the lack of gene encoding the cytochrome b6/f complex in the *T. amphioxeia* plastid genome^25^. Two *PetH* isoforms were identified as having originated from peridinin and fucoxanthin dinoflagellates (Fig. 3c). In *D. fotii*, the transcription level of the *PetH* isoform originating from fucoxanthin dinoflagellates was significantly higher than that of the *PetH* isoform originated from peridinin dinoflagellates (Fig. 3c). Thus, in *D. fortii*, the *PetH* isoform originating from peridinin dinoflagellates may have been replaced by that originating from fucoxanthin dinoflagellates. Furthermore, evolutionary convergence does not appear to have occurred between the two isoforms of this gene in *D. fortii*. Finally, *PsbO* was estimated to have originated from haptophytes (Fig. 3d).

**Figure 4:**
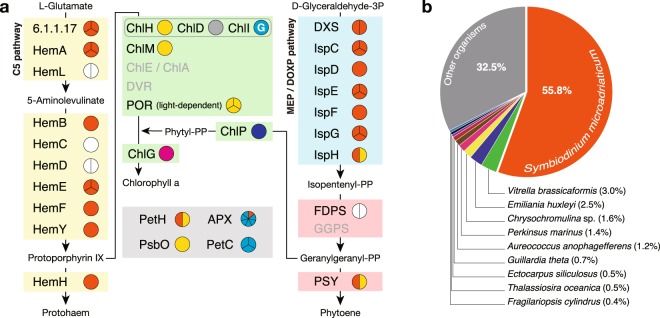
(**a**) The deduced origins of proteins involved in porphyrin, chlorophyll, isoprenoid and carotenoid biosyntheses as well as in photosynthesis, and (**b**) the top 10 species in the Basic Local Alignment Search Tool (BLAST) search are shown. Yellow, green, blue, pink and grey boxes in (**a**) indicate proteins related to the biosynthesis pathways for porphyrins (haem), chlorophylls, isoprenoids and carotenoids, as well as photosynthesis, respectively. Names presented in black and grey indicate identified and unidentified proteins in this study, respectively. Pie charts present identified proteins, and the colours denote proteins originated from peridinin dinoflagellates (orange), fucoxanthin dinoflagellates (yellow), haptophytes (blue), chlorarachniophytes (magenta), cryptophytes (light blue) and cyanobacteria (grey). White pie charts indicate proteins for which the origin is unclear due to a low phylogenetic tree resolution. ‘G’ in the ChlI pie chart indicates that the *ChlI* gene was coded in the chloroplast genome of *T. amphioxeia* (accession no. YP_009159192). In (**b**), homologous species in the BLAST search are arranged by relative abundance in descending order in a clockwise direction.

**Figure 5:**
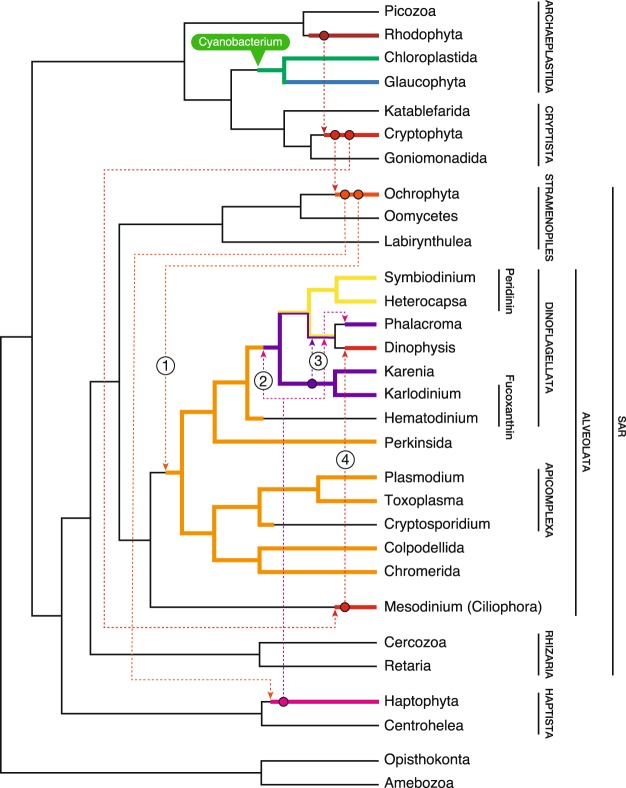
Schematic illustration of the proposed mechanism of plastid acquisition in *Dinophysis*. The model proposed by Bodył (2018)^2^ was modified. (1) A common ancestor of dinoflagellates, perkinsids, apicomplexans, colpodellids and chromerids acquired plastids from Ochrophyta. (2) The ancestor of peridinin dinoflagellates acquired a haptophyte plastid and evolved into both the peridinin and fucoxanthin dinoflagellates that exist today. (3) *Dinophysis* retained the original haptophyte plastid or evolved a peridinin plastid and then acquired a new plastid from either haptophytes or Kareniaceae, which possess the original haptophyte plastid. *Dinopysis* subsequently evolved into two lineages: a lineage that primarily possessed the haptophyte plastid, e.g. *Phalacroma mitra*, and (4) a lineage that switched from using a haptophyte to using a cryptophyte plastid via *Mesodinium*.

Our findings indicated that in *Dinophysis*, the genes involved in porphyrin, chlorophyll and isoprenoid biosyntheses as well as in photosynthesis are acquired from fucoxanthin dinoflagellates, haptophytes, chlorarachniophytes, cyanobacteria and cryptophytes via LGT (Fig. 4a). Furthermore, the *D. fortii* genome may harbour other proteins encoded by genes acquired via LGT because approximately half of the analysed proteins were homologues of proteins of the peridinin dinoflagellate *Symbiodinium microadriaticum*, whereas the remainder were homologues of proteins of other organisms, particularly haptophytes (2.5% of *Emiliania huxleyi* and 1.6% of *Chrysochromulina* sp.; Fig. 4b). In contrast, we obtained very little evidence of LGT from cryptophytes (0.7% of *Guillardia theta*, Fig. 4b). These results suggest a close relationship between ancestral *Dinophysis* spp. and haptophytes and/or fucoxanthin dinoflagellates during the course of evolution. Conventionally, the phagocytotic digestion of other organisms has been considered the driving force for the acquisition of genes from other organisms (according to the ‘you are what you eat’ ratchet model^26^). Reportedly, *D. fortii* possesses digestive food vacuoles in their body^27^. In addition, our results indicate that the target genes in *Dinophysis* were derived from various organisms. Therefore, the major LGT events likely occurred within the common ancestors of *Dinophysis* spp., and their close relationships with symbionts accelerated gene flow, as illustrated in the ‘shopping bag’ model^28^. Once the ancestral species of *Dinophysis* began engulfing or living in the proximity of haptophytes and/or fucoxanthin dinoflagellates, the peridinin plastid may have reduced along with the gene flow to the *Dinophysis* genome from the potential symbionts. *Phalacroma mitra* belonging to a sister linage of *Dinophysis*^29^ predominantly derived kleptoplastids from haptophytes and may have continued to derive these even after the species diverged. Conversely, although plastids of peridinin dinoflagellate origin are generally considered to have been derived from red algae, some studies have postulated these to have been derived from haptophytes^2,30^. Moreover, the heterotrophic dinoflagellate *Pfiesteria piscicida* has been reported to harbour genes derived from fucoxanthin dinoflagellates^31^. Based on this evidence, we suggest that the genes derived from fucoxanthin dinoflagellates and/or haptophytes have been either vertically inherited from the ancestor of dinoflagellates and/or horizontally transferred from haptophytes as in fucoxanthin dinoflagellates. Nonetheless, in the present study, since the major genes acquired via LGT originated from haptophytes and/or fucoxanthin dinoflagellates, the relationship between ancestral *Dinophysis* and haptophytes and/or fucoxanthin dinoflagellates may have remained steady. Since such kleptoplastids were not permanently retained in *Dinophysis*, its ancestors may have been required to continue feeding on other organisms to derive plastids. Consequently, LGT may have occurred from various organisms such as cyanobacteria and chlorarachniophytes (Fig. 1b, e). Because the extant *Dinophysis* spp. feed on other potential prey organisms in addition to *M. rubrum*^32^, LGT from other organisms is possible in these species. However, this scenario is only an evolutionary hypothesis (Fig. 5) and remains to be discussed in the light of further evidence and other speculations. During the course of evolution of kleptoplastids in *Dinophysis* from the time when they began feeding on *M. rubrum* and utilising the derived plastid, an evolutionary transition towards the retention of plastids obtained from cryptophytes may have begun before the plastids of haptophyte origin were established.

## Methods

### Establishment of clonal strains

Samples of the ciliate *M. rubrum* and the cryptophyte *T. amphioxeia* were isolated from Inokushi Bay in Oita Prefecture, Japan, at the end of February 2007^33^. Cultures were established as prey for *D. fortii* in modified f/2 medium^34,35^ with a salinity of 30 practical salinity units (psu) and maintained at 18 °C under a photon irradiance of 100 µmol·m^−2^·s^−1^ provided by cool-white fluorescent lamps under a 12:12 h light:dark cycle. *T. amphioxeia* subculture was maintained by re-inoculating 0.1–0.3 mL of culture (ca. 1.2 × 10^5^ cells mL^−1^) into fresh modified f/2 medium once per week, and *M. rubrum* subculture was maintained by re-inoculating 40 mL of culture (2,000–3,000 cells mL^−1^) into 110 mL of fresh modified f/2 medium and 60 µL of the *T. amphioxeia* culture (ca. 1.2 × 10^5^ cells mL^−1^) in 250-mL polycarbonate Erlenmeyer flasks once per week.

*D. fortii* culture was established from a seawater sample collected from Saloma Lake, Japan (143°148′E, 44°15′N) in October 2015. Cells were picked up by micropipetting and rinsed several times in filtered seawater (0.22 µm). The established *D. fortii* culture was maintained by re-inoculating 15 mL of culture (500–1,000 cells mL^−1^) into 150 mL of *M. rubrum* culture (containing 500–1,000 cells mL^−1^; 1:4 dilution of fresh culture medium) in 250-mL polycarbonate Erlenmeyer flasks once every 3 weeks under the same conditions as outlined above.

### Culture conditions for RNA sequencing

Once established, the clonal strains of *T. amphioxeia*, *M. rubrum* and *D. fortii* were maintained in 150 mL of culture solution at 18 °C under photon irradiances of 100 or 20 µmol·m^−2^·s^−1^ provided by cool-white fluorescent lamps under a 12:12 h light:dark cycle.

At the start of the experiment, *M. rubrum* cultures were transferred *D. fortii* cultures (in duplicate) at a predator:prey ratio of 1:10, thus allowing *D. fortii* to acquire and retain plastids from *M. rubrum*. After 5 days, *D. fortii* cultures were filtered through a 20-µm nylon mesh to remove any remaining *M. rubrum* cells, and the filtered culture media were filtered again through 8-µm polycarbonate filters (GE Healthcare, Tokyo, Japan). *D. fortii* cells were then re-inoculated into culture media devoid of prey and incubated for 1 week. Thereafter, *D. fortii* cells were once again trapped by filtering the media through a 20-µm nylon mesh and collected by centrifugation at 5,000 × g for 2 min. The cells were immediately immersed in RNALater (Thermo Fisher Scientific, Waltham, MA, USA), left overnight at 4 °C and stored at −80 °C until further use.

*T. amphioxeia* and *M. rubrum* sequences from the *D. fortii* RNA sequences, *T. amphioxeia* and *M. rubrum* cells maintained under the highest photon irradiance, followed by 30 min in the dark, were removed from the cultures using 1-µm polycarbonate filters. RNALater was applied to each filter for 5 min to preserve the total RNA. After removing RNALater, the filters were stored at −80 °C until further use.

### RNA extraction and cDNA library construction

Total RNA was extracted from *T. amphioxeia*, *M. rubrum* and *D. fortii* preserved in RNALater and stored at –80 °C using the TRIzol Plus RNA Purification Kit (Thermo Fisher Scientific); any contaminating DNA was digested using PureLink DNase (Thermo Fisher Scientific), according to the manufacturer’s instructions. The concentration and purity total RNA were determined using a Qubit RNA HS Assay Kit (Thermo Fisher Scientific) and a Bioanalyzer 2100 with RNA 6000 Nano Kit (Agilent Technologies, Inc., Santa Clara, CA, USA), according to the manufacturer’s instructions.

Duplicate libraries for *D. fortii* under the two photon irradiance conditions and one library each for *T. amphioxeia* and *M. rubrum* under the highest photon irradiance and dark conditions were constructed from the total RNA (1 µg) in accordance with the TruSeq RNA Sample Prep ver. 2 (LS) protocol (Illumina, Inc.). Complementary DNA (cDNA) was synthesised using SuperScript III reverse transcriptase (Thermo Fisher Scientific). The cDNA libraries were sequenced into 150-bp paired-end reads using NextSeq 500 (Illumina, Inc.) at the Research Center for Bioinformatics and Biosciences of the National Research Institute of Fisheries Science, Yokohama, Japan.

### Sequence analysis

Sequences from individual samples were generated using the bcl2fastq pipeline ver. 2.17 (Illumina, Inc.). Any adapter sequences, low-quality ends (<QV30) and unpaired reads were removed from the sequences using Trimmomatic^36^. The sequence length and the quality of the remaining reads were confirmed using FastQC^37^, then the remaining paired-end reads were assembled using Trinity^18^ using the ‘-min_kmer_cov = 2′ command option and under default settings for all other options. ORFs of >300 bp were extracted from the assembled sequences and translated into amino acid sequences using TransDecoder^38^. ORFs of 95% homologous amino acid sequences were clustered using the CD-HIT programme^39^ using the ‘–c 0.95′ command option and under default settings for all other options to remove redundant amino acid sequences. The remaining amino acid sequences were searched against those of *T. amphioxeia* and *M. rubrum* using the Protein Basic Local Alignment Search Tool (BLASTP) programme, with a threshold of sequence homology of >98% identity to remove the amino acid sequences of the prey species.

Proteins derived from *D. fortii* were annotated based on their homology to sequences in the nr database of NCBI and the UniRef90 database^40^, using the MMseqs2 programme^41^, with a threshold e-value of <1e^–3^. GO numbers, which are shared with the accession numbers used in UniRef90^40,42^, were assigned from the best hits of the MMseqs2 results against UniRef90. EC numbers were obtained from GO numbers using the Blast2GO software^43^ to identify proteins related to porphyrin and chlorophyll metabolism, terpenoid backbone biosynthesis and photosynthesis.

### Comparison of transcription levels among isoforms

Transcription levels of each gene were determined based on the number of mapped reads. The reads were mapped to each gene using Bowtie2^44^ and counted using RSEM^45^. Transcription levels were normalised among the libraries using the trimmed mean of M-values method^46^ with the edgeR package^47^ in R software ver. 3.3.1^48^. The normalised fragments per kilobase per million mapped fragments (FPKM) of different isoforms were statistically compared using ANOVA and Student’s *t*-test with R software ver. 3.3.1^48^.

### Phylogenetic analysis

Amino acid sequences of several organisms, including Viridiplantae, Rhodophyta, Stramenopiles, Haptophyta, Cryptophyta, Chromerida, Chlorarachniophyta and Dinoflagellates were obtained from public databases (Supplementary Tables 3 and 4). Protein sequences several organisms were retrieved based on their homology to the target proteins of *D. fortii* using the BLASTP programme. Multiple sequence alignments were performed using MAFFT ver. 7.212^49^, and gaps were automatically trimmed by trimAl^50^ using the ‘–automated1′ command option and under default settings for all other options. The best-fit evolutionary model for each alignment was identified by ModelFinder^51^ using the Akaike information criterion (Supplementary Table 4) and subjected to the maximum-likelihood (ML) and Bayesian phylogenetic analyses. ML trees were inferred using RAxML ver. 8.2.4^52^ with 100 bootstrap replicates, while the posterior probabilities of nodes in ML trees were calculated with MrBayes ver. 3.1.2^53^ using a Metropolis-coupled Markov chain Monte Carlo procedure starting from a random tree and sampled every 100 generations for a total of 1 million generations. One heated and three cold chains were simultaneously started, and the best fitting substitution model for each protein set was used for analyses. The initial 25% of the sampled trees were discarded as ‘burn in’ prior to the construction of the consensus phylogeny.

## Supplementary information

Supplementary info

Supplementary dataset
