# Supplementary material for: Genes functioned in kleptoplastids of Dinophysis are derived from haptophytes rather than from cryptophytes
Source: Sci Rep. 2019 Jun 21;9:9009. doi: 10.1038/s41598-019-45326-5 (PMC6588620; doi:10.1038/s41598-019-45326-5)
Supplement: Supplementary file 1 — Supplementary info [file 41598_2019_45326_MOESM1_ESM.pdf]

Supplementary Information

Genes functioned in kleptoplastids of *Dinophysis* are derived from haptophytes rather than from cryptophytes.

Yuki Hongo[1,*], Akinori Yabuki[2], Katsunori Fujikura[2], Satoshi Nagai[1]

[1]Department of Research Center for Bioinformatics and Biosciences, National Research Institute of Fisheries Science, Japan Fisheries Research and Education Agency, 2-12-4 Fukuura, Kanazawa, Yokohama, Kanagawa 236-8648, Japan

[2]Department of Marine Biodiversity and Environmental Assessment Research Center, Japan Agency for Marine-Earth Science and Technology, 2-15 Natsushima-cho, Yokosuka, Kanagawa 237-0061, Japan

***Correspondence:** hongoy@affrc.go.jp

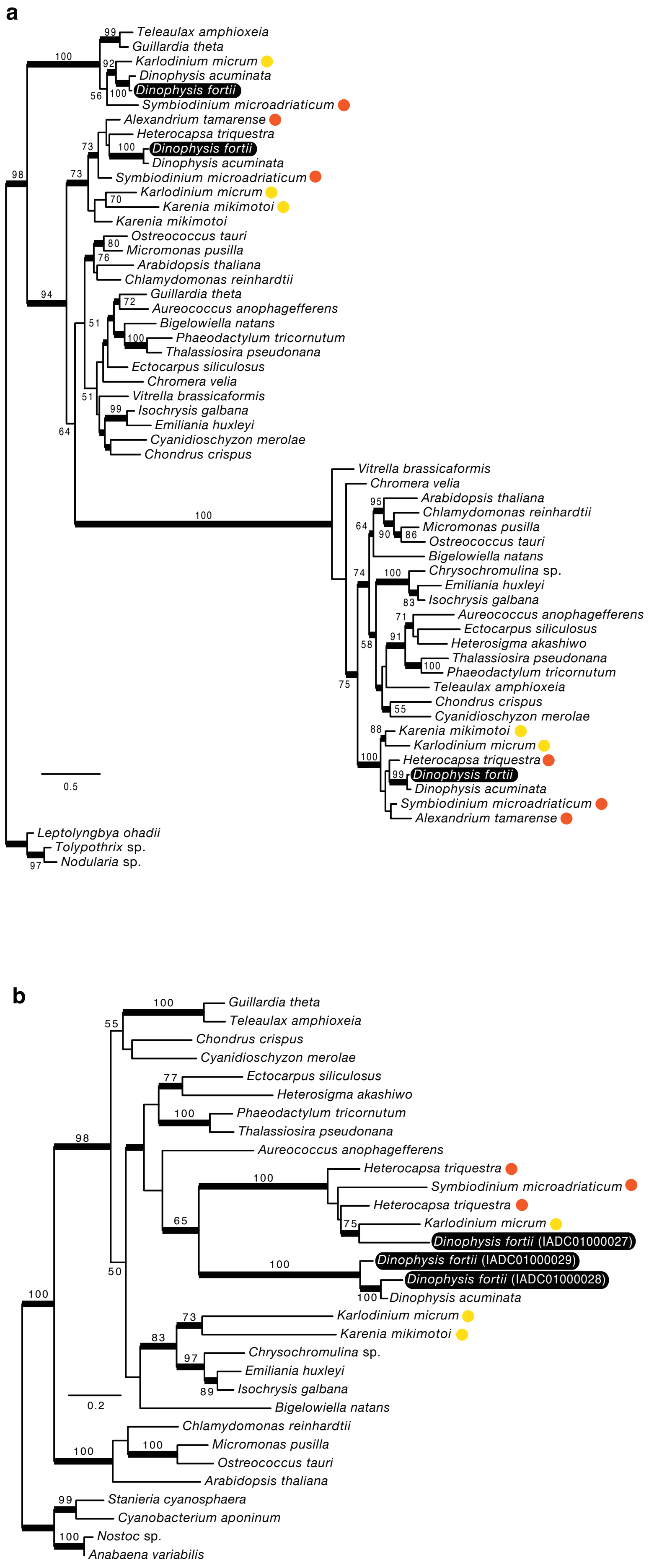

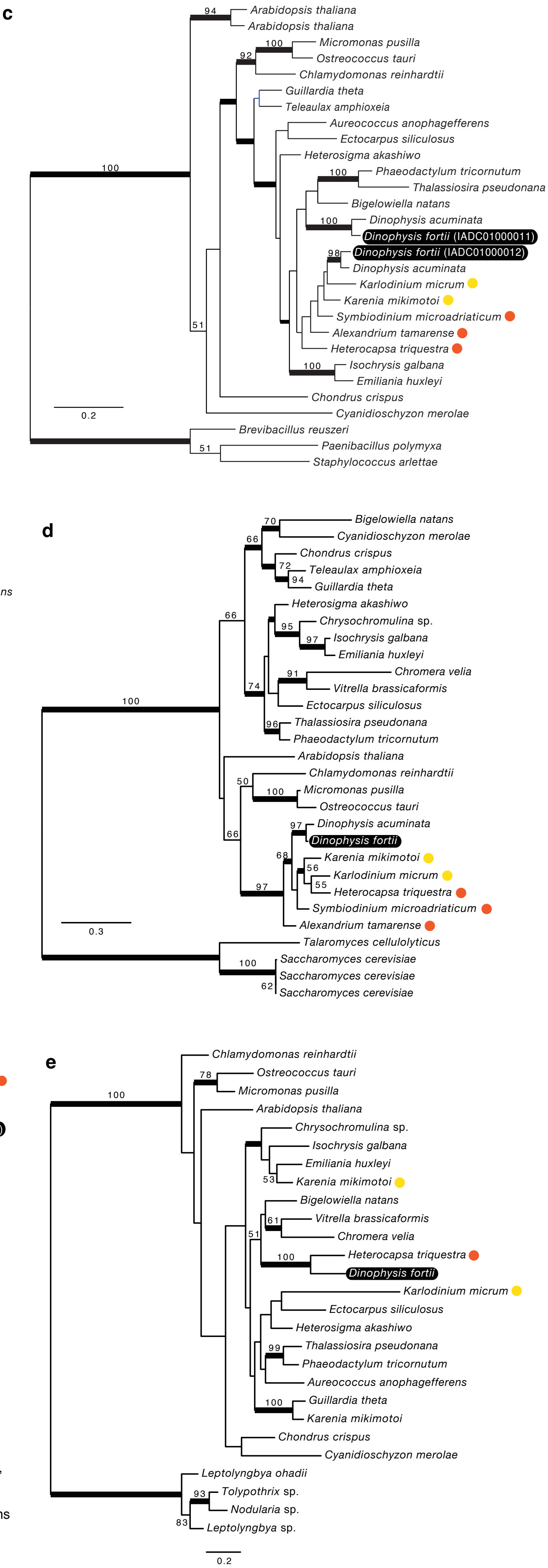

Figure S1. Maximum-likelihood tree of (a) Glu-tRNA ligase, (b) HemA, (c) HemL, (d) HemB, and (e) HemC. Bootstrap values ≥50% are shown. Nodes supported by Bayesian posterior probabilities ≥0.95 are highlighted with bold lines. Coloured circles indicate proteins of peridinin dinoflagellates (orange), fucoxanthin dinoflagellates (yellow). Proteins of Dinophysis fortii are presented on black background with white font.

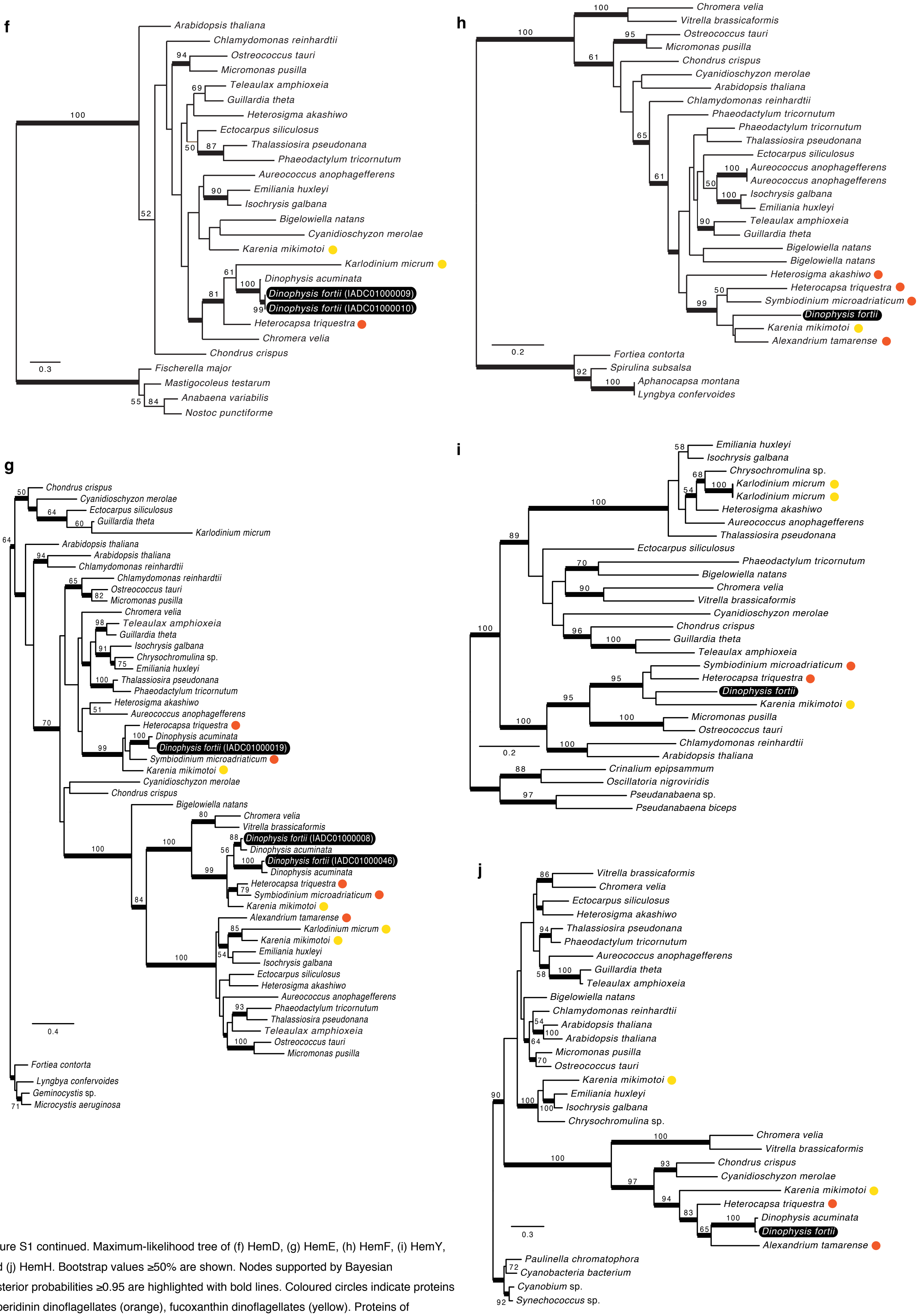

Figure S1 continued. Maximum-likelihood tree of (f) HemD, (g) HemE, (h) HemF, (i) HemY, and (j) HemH. Bootstrap values ≥50% are shown. Nodes supported by Bayesian posterior probabilities ≥0.95 are highlighted with bold lines. Coloured circles indicate proteins of peridinin dinoflagellates (orange), fucoxanthin dinoflagellates (yellow). Proteins of Dinophysis fortii are presented on black background with white font.

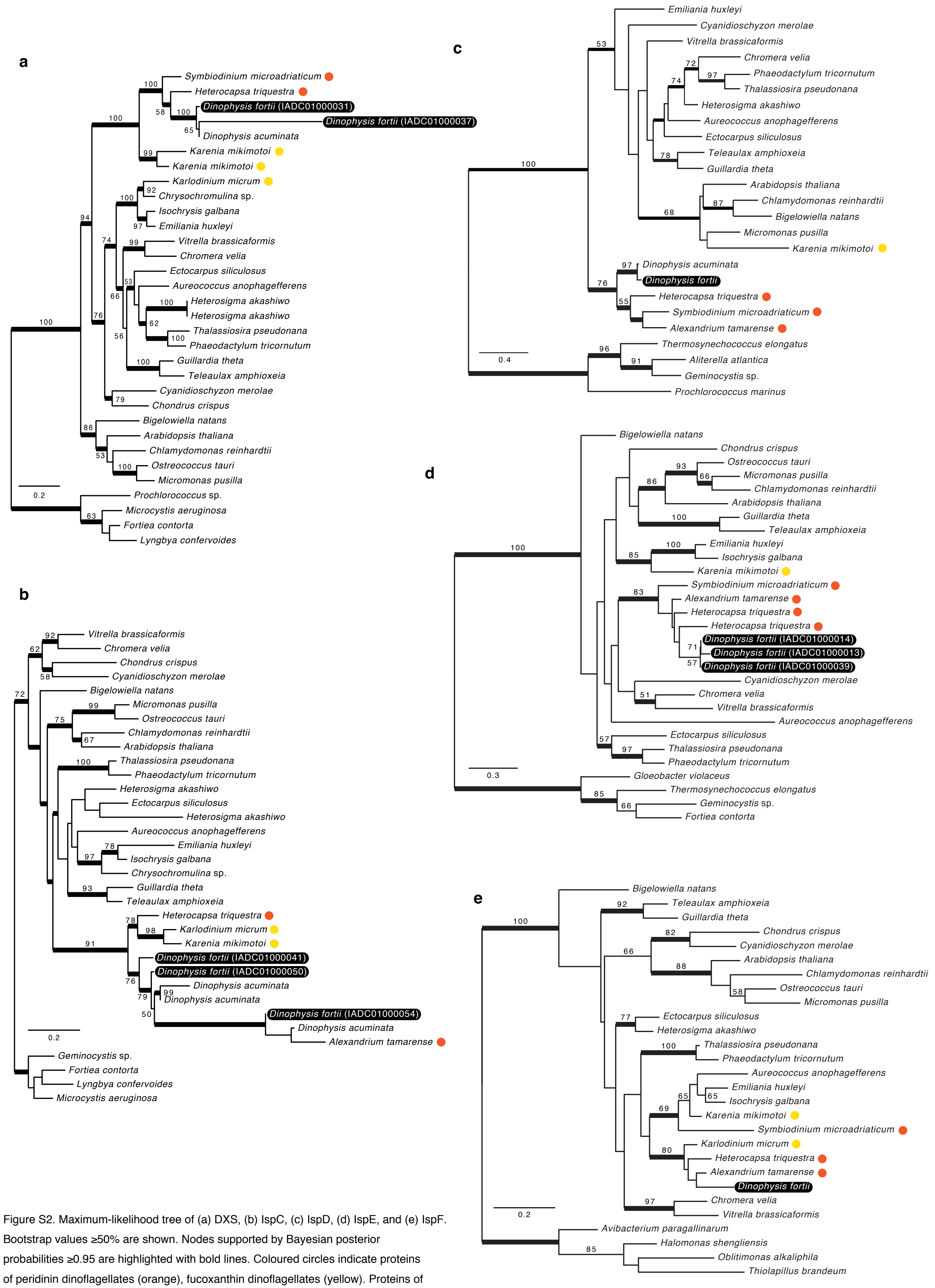

Figure S2. Maximum-likelihood tree of (a) DXS, (b) IspC, (c) IspD, (d) IspE, and (e) IspF. Bootstrap values ≥50% are shown. Nodes supported by Bayesian posterior probabilities ≥0.95 are highlighted with bold lines. Coloured circles indicate proteins of peridinin dinoflagellates (orange), fucoxanthin dinoflagellates (yellow). Proteins of Dinophysis fortii are presented on black background with white font.

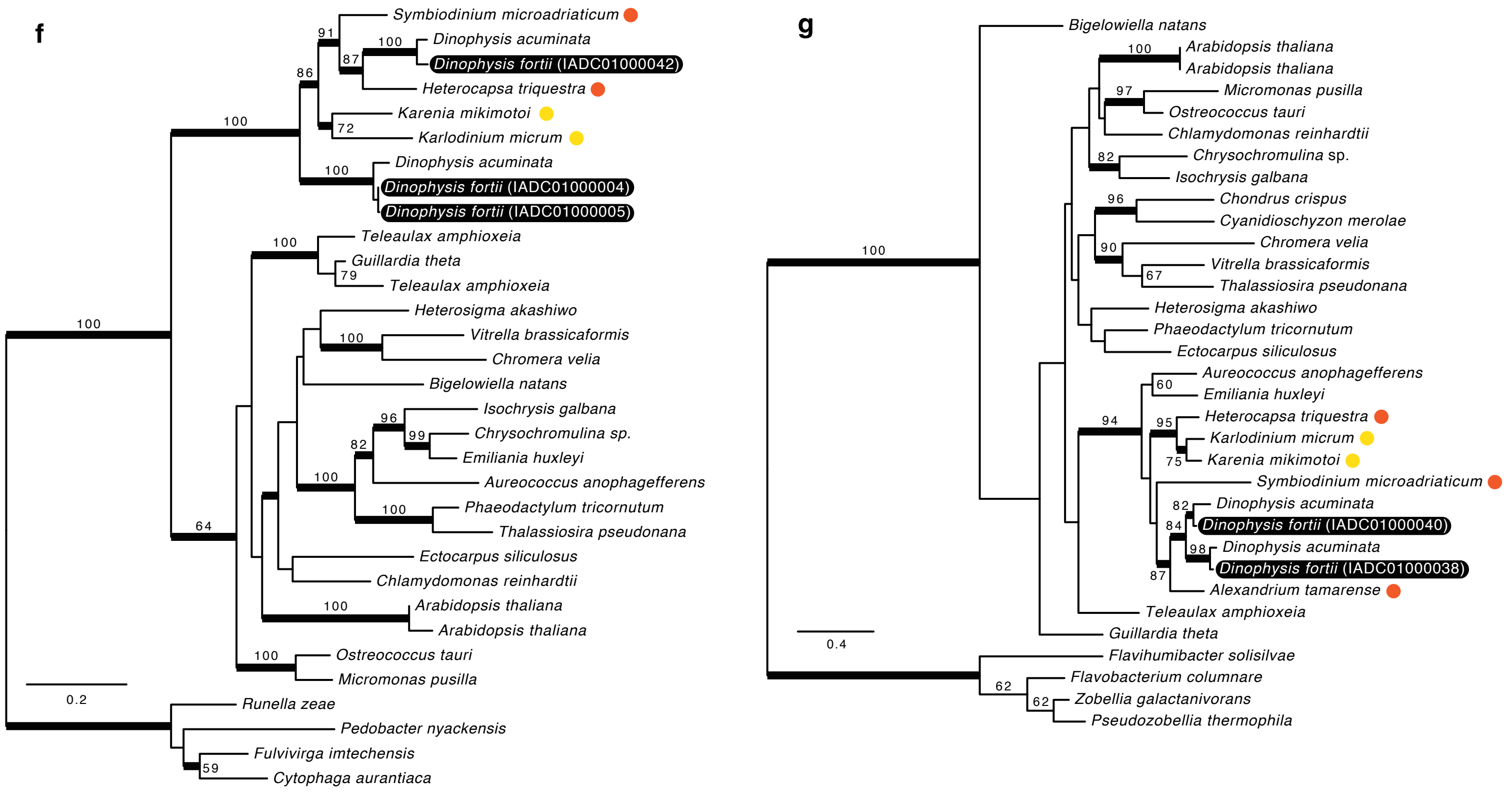

Figure S2 continued. Maximum-likelihood tree of (f) IspG, and (g) FBPS. Bootstrap values ≥50% are shown. Nodes supported by Bayesian posterior probabilities ≥0.95 are highlighted with bold lines. Coloured circles indicate proteins of peridinin dinoflagellates (orange), fucoxanthin dinoflagellates (yellow). Proteins of Dinophysis fortii are presented on black background with white font.ß

Supplementary table 1. Summary of sequence assembly, extracting ORF and homology search

	Teleaulax amphioxeia	*Mesodinium rubrum*	*Dinophysis fortii*
Assembly			
Number of contigs	132,239	144,278	217,120
Total bases (bp)	108,910,558	111,494,090	166,169,937
Longest contig length (bp)	20,618	13,393	27,058
Shortest contig length (bp)	201	201	201
Average of contig length (bp)	824	773	765
N50	1,314	1,147	1,194
Extraction of ORFs			
Number of ORFs*			372,783 (119,098)
Homology search**			
Nr			59,907
Uniref90			61,878
GO numbers			39,850
EC numbers			10,328

N50, minimum contig length such that the sum of contigs of equal length or longer is at least 50% of the total length of all contigs.

*, Number of amino acid sequences. Number of nucleotide sequences showed in parentheses.

**, Numbers showed amino acid sequences.

Supplementary table 2. Accession numbers of identified proteins.

	Protein (gene) name	Accession number	Gene origin
Porphyrin and chlorophyll biosynthesis	Glutamate-tRNA ligase	IADC01000051	Peridinin type
		IADC01000052	Peridinin type
		IADC01000044	Peridinin type
	Glutamyl-tRNA reductase (*hemA*)	IADC01000027	Peridinin type
		IADC01000028	Peridinin type
		IADC01000029	Peridinin type
	Glutamate-1-semialdehyde 2,1-aminomutase (*hemL*)	IADC01000011	?
		IADC01000012	?
	Delta-aminolevulinate dehydratase (*hemB*)	IADC01000056	Peridinin type
	Hydroxymethylbilane synthase (*hemC*)	IADC01000015	?
	Uroporphyrinogen-III synthase (*hemD*)	IADC01000009	?
		IADC01000010	?
	Uroporphyrinogen decarboxylase (*hemE*)	IADC01000008	Peridinin type
		IADC01000019	Peridinin type
		IADC01000046	Peridinin type
	Coproporphyrinogen oxidase (*hemF*)	IADC01000043	Peridinin type
	Protoporphyrinogen oxidase (*hemY*)	IADC01000049	Peridinin type
	Ferrochelatase (*hemH*)	IADC01000021	Peridinin type
	Magnesium-protoporphyrin IX chelatase (*chlH*)	IADC01000035	Fucoxanthin type
	Magnesium-protoporphyrin IX chelatase (*chlD*)	IADC01000057	Bacteria
	Magnesium protoporphyrin IX methyltransferase (*chlM*)	IADC01000058	Fucoxanthin type
	Protochlorophyllide oxidoreductase (*por*)	IADC01000023	Fucoxanthin type
		IADC01000022	Fucoxanthin type
		IADC01000047	Fucoxanthin type
	Chlorophyll synthase (*chlG*)	IADC01000048	Chlorarachniophyte
Terpenoid biosynthesis	1-deoxy-D-xylulose-5-phosphate synthase (*dxs*)	IADC01000031	Peridinin type
		IADC01000037	Peridinin type
	1-deoxy-D-xylulose-5-phosphate reductoisomerase (*ispC*)	IADC01000050	Peridinin type
		IADC01000041	Peridinin type
		IADC01000054	Peridinin type
	2-C-methyl-D-erythritol 4-phosphate cytidylyltransferase (*ispD*)	IADC01000055	Peridinin type
	4-(cytidine 5'-diphospho)-2-C-methyl-D-erythritol kinase (*ispE*)	IADC01000014	Peridinin type
		IADC01000039	Peridinin type
		IADC01000013	Peridinin type
	2-C-methyl-D-erythritol 2,4-cyclodiphosphate synthase (*ispF*)	IADC01000020	Peridinin type
	4-hydroxy-3-methylbut-2-en-1-yl diphosphate synthase (*ispG*)	IADC01000042	Peridinin type
		IADC01000004	Peridinin type
		IADC01000005	Peridinin type
	4-hydroxy-3-methylbut-2-en-1-yl diphosphate reductase (*ispH*)	IADC01000045	Fucoxanthin type
		IADC01000002	Peridinin type
	Farnesyl-diphosphate synthase (*fdps*)	IADC01000040	?
		IADC01000038	?
	Phytoene synthetase (*psy*)	IADC01000030	Fucoxanthin type
		IADC01000053	Peridinin type
Protein functioned in chloroplast	Oxygen-evolving enhancer protein (*psbO*)	IADC01000036	Fucoxanthin type
	Ascorbate peroxidase (*apx*)	IADC01000017	Cryptophyte
		IADC01000003	Cryptophyte
		IADC01000006	Cryptophyte
		IADC01000007	Cryptophyte
		IADC01000016	Cryptophyte
		IADC01000018	Cryptophyte
		IADC01000026	Peridinin type
	Cytochrome b6/f complex iron-sulfur subunit (*petC*)	IADC01000034	Cryptophyte
		IADC01000032	Cryptophyte
		IADC01000033	Cryptophyte
	Ferredoxin-NADP(+) reductase (*petH*)	IADC01000001	Peridinin type
		IADC01000025	Fucoxanthin type
	geranylgeranyl reductase (*chlP*)	IADC01000024	Haptophyte

Supplementary table 3. Retrived sequence data from public database.

Bigelowiella natans	MMETSP0045	Chlorarachniophyta
Chromera velia	MMETSP0290	Chromerida
Vitrella brassicaformis	MMETSP1451	Chromerida
Guillardia theta	GCF_000315625.1_Guith1_protein	Cryptophyta
Teleaulax amphioxus	in this study	Cryptophyta
*Karenia mikimotoi**	DRR028933, DRR028934	Alveolata
Prorocentrum micans	MMETSP0251	Alveolata
Alexandrium tamarense	MMETSP0378	Alveolata
Heterocapsa triquestra	MMETSP0448	Alveolata
Dinophysis acuminata	MMETSP0797	Alveolata
Karlodinium micrum	MMETSP1015	Alveolata
Symbiodinium microadriaticum	GCA_001939145.1_ASM193914v1_protein	Alveolata
Chlamydomonas reinhardtii	GCF_000002595.1_v3.0_protein	Viridiplantae
Micromonas pusilla	GCF_000151265.2_CCMP1545_v2.0_protein	Viridiplantae
Ostreococcus tauri	GCF_000214015.2_version_050606_protein	Viridiplantae
Arabidopsis thaliana	GCF_000001735.3_TAIR10_protein	Viridiplantae
Emiliania huxleyi	GCF_000372725.1_Emiliana_huxleyi_CCMP1516_main_genome_assembly_v1.0_protein	Haptophyta
Isochrysis galbana	MMETSP0595	Haptophyta
Chrysochromulina sp. CCMP291	GCA_001275005.1_Ctobinv2_protein	Haptophyta
Ectocarpus siliculosus	GCA_000310025.1_ASM31002v1_protein	Stramenopiles
Thalassiosira pseudonana	GCF_000149405.2_ASM14940v2_protein	Stramenopiles
Phaeoductylum tricornutum	GCF_000150955.2_ASM15095v2_protein	Stramenopiles
Aureococcus anophagefferens	GCF_000186865.1_v_1.0_protein	Stramenopiles
Heterosigma akashiwo	MMETSP0292	Stramenopiles
Cyanidioschyzon merolae	GCF_000091205.1_ASM9120v1_protein	Rhodophyta
Chondrus crispus	GCF_000350225.1_ASM35022v2_protein	Rhodophyta

*Low-quality ends (<QV30) and unpaired reads were removed using Trimmomatic (Bolger et al., 2014) and then assembled by Trinity (Grabherr et al., 2011) with the '-min_kmer_cov=2' command option and the other options were set to default.
